# Curcumin Analog GO‐Y030 Triggers JNK and p38 Signalling to Activate Apoptotic Cascades in Human Osteosarcoma Cells

**DOI:** 10.1111/jcmm.70383

**Published:** 2025-02-13

**Authors:** Yu‐Hsien Lin, Jia‐Sin Yang, Chia‐Hsuan Chou, Tzu‐Yu Huang, Shun‐Fa Yang, Ko‐Hsiu Lu

**Affiliations:** ^1^ Institute of Medicine Chung Shan Medical University Taichung Taiwan; ^2^ Division of Spinal Surgeries, Department of Orthopedic Surgery Taichung Veteran General Hospital Taichung Taiwan; ^3^ Department of Medical Research Chung Shan Medical University Hospital Taichung Taiwan; ^4^ Department of Orthopedics Chung Shan Medical University Hospital Taichung Taiwan; ^5^ School of Medicine Chung Shan Medical University Taichung Taiwan

**Keywords:** apoptosis, curcumin, GO‐Y030, JNK, osteosarcoma, p38

## Abstract

Osteosarcoma, the most common primary bone cancer in adolescents, often carries a grim prognosis due to its high metastatic potential. Due to its low bioavailability, curcumin limits its adjuvant efficacy in improving prognosis and long‐term survival in osteosarcoma patients. To investigate apoptosis induced by the synthesised curcumin analog GO‐Y030 in human osteosarcoma cells, flow cytometry, annexin V‐fluorescein isothiocyanate‐labelled/propidium iodide staining, human apoptosis array, and Western blotting were used. GO‐Y030 dose‐dependently reduced viability and induced sub‐G1 arrest and apoptosis in human osteosarcoma U2OS and 143B cells. GO‐Y030 significantly activated caspases 8, 9, and 3, while suppressing cellular inhibitors of apoptosis protein 1 (cIAP‐1) and X‐chromosome‐linked IAP. GO‐Y030 increased the phosphorylation of extracellular signal‐regulated protein kinases (ERK)1/2, c‐Jun N‐terminal kinases (JNK)1/2, and p38. Inhibitors of JNK (JNK‐IN‐8) and p38 (SB203580) suppressed GO‐Y030‐induced cleavage of caspases 8, 9, and 3, whereas co‐treatment with the ERK inhibitor (U0126) did not lessen their activation. Overall, GO‐Y030 triggers both extrinsic and intrinsic apoptotic cascades in U2OS and 143B cells by activating the JNK1/2 and p38 pathways, shedding light on its mechanism of action against human osteosarcoma cells.

## 1 Introduction

Curcumin (diferuloylmethane), a polyphenolic compound of diet constituents found in turmeric, was initially identified in the rhizome of *Curcuma longa* Linn, an East Indian plant [1, 2]. Despite interference with nuclear factor κ‐light‐chain‐enhancer of activated B cells (NF‐κB), β‐catenin, and oncogenes, it exhibits potent anti‐carcinogenic effects by suppressing tumour growth and blocking growth factor signalling [3]. Also, it possesses anti‐invasive, antimetastatic, and antiangiogenic properties [4, 5, 6, 7]. Clinical trials found that daily oral curcumin (3.6 g/kg) positively impacted cancer patients' therapy [8, 9]. Despite being well‐tolerated with minimal toxicity, its clinical utility is hindered by challenges such as low aqueous solubility, poor absorption, restricted tissue distribution, rapid metabolism, and quick elimination from the body, leading to limited availability for chemotherapeutic applications in cancer patients, including osteosarcoma [1, 2].

Osteosarcoma, the primary bone malignancy most commonly affecting adolescents and young adults, exhibits a high potential for metastasis to the lungs and leads to significant treatment failure and mortality [10, 11]. Historically, surgical resection or amputation was the mainstay of treatment, yet despite these efforts, early pulmonary metastasis was prevalent [12, 13]. Recent advancements combining surgery with chemotherapy, tailored to radiological staging and modern protocols, have improved long‐term survival rates to around 68% [14, 15]. However, inducing apoptosis and other cell death and mitigating metastasis remain critical for developing more effective therapeutic strategies against this aggressive cancer [16].

Unlike necrosis, which results from acute cell injury, apoptosis is programmed cell death [12, 17]. Apoptosis is initiated irreversibly and proceeds through the intrinsic (mitochondria) pathway triggered by internal cellular stress or the extrinsic (receptor) pathway responding to external signals from nearby signalling. Both pathways involve caspases—proteases that degrade proteins—to execute cell death. Initiator caspases 8 or 9 activate executioner caspases 3 or 7, which degrade cellular proteins indiscriminately. Stress‐inducible molecules like mitogen‐activated protein kinase (MAPK)/extracellular signal‐regulated protein kinases (ERK), c‐Jun N‐terminal kinase (JNK), p38, and NF‐κB are involved in transmitting apoptotic cascades [18, 19]. Inhibitors of apoptosis proteins (IAPs), such as cellular IAP‐1 and ‐2 (cIAP‐1 and cIAP‐2) and X‐linked IAP (XIAP), suppress both intrinsic and extrinsic signals of cell apoptosis.

Through an extensive screening process aimed at identifying the structural elements responsible for curcumin's anti‐cancer properties and developing improved analogs, GO‐Y030, was synthesised by Ohori et al. [20]. Initial evaluations showed that GO‐Y030 effectively suppressed growth in multiple cancer cell lines, demonstrating 8–40 times inhibition than curcumin. By inhibiting glycolysis in melanoma cells, GO‐Y030 inhibits lung metastasis in mouse models [21]. Importantly, it maintains curcumin's favourable safety profile [22, 23]. While GO‐Y030 shows promise as a potential adjuvant agent in several cancers, such as colorectal [22], breast [22, 24], pancreatic [24, 25], thyroid [25, 26], and cholangiocarcinoma [25] due to its potency and bioavailability, its effects on human osteosarcoma apoptosis need to be elucidated.

## 2 Methods and Materials

### 2.1 Cell Culture and GO‐Y030 Treatment

Human osteosarcoma U2OS and 143 B cells were obtained from the Food Industry Research and Development Institute (Hsinchu, Taiwan) and cultured in Dulbecco's Modified Eagle Medium (DMEM; Gibco‐BRL, Gaithersburg, MD, USA). They were supplemented with 10% fetal bovine serum (FBS; Hyclone Laboratories Inc., Logan, UT, USA) and 1% penicillin (100 U/mL)/streptomycin (100 μg/mL) (Sigma‐Aldrich, St. Louis, MO, USA). U2OS and 143B cells, plated in 24‐well plates, were added at different concentrations (0.5 μM, 1 μM, 2 μM, 4 μM, and 8 μM) of GO‐Y030 (Tokyo Chemical Industry Co. Ltd.) for 24 h for the subsequent assays. They were maintained in a humidified atmosphere of 5% CO2 incubator at 37°C, as previously described [27, 28].

### 2.2 Microculture Tetrazolium (MTT) Assay

Using MTT assay, we examined the cytotoxicity of GO‐Y030 at different concentrations (0.5 μM, 1 μM, 2 μM, 4 μM, and 8 μM). After treatment and removal of the media, the cells were washed with phosphate‐buffered saline (PBS). Then, U2OS (6.0 × 10^4^ cells/dish) and 143B (9.0 × 10^5^ cells/dish) cells were incubated with microculture tetrazolium (0.5 mg/mL) for 4 h, as previously described [29, 30].

### 2.3 Flow Cytometry

Due to the direct proportion to formazan production, the viable cell number per dish can be measured spectrophotometrically at 563 nm following solubilisation with isopropanol after annexin V‐fluorescein isothiocyanate‐labelled (FITC) and propidium iodide (PI) staining. Flow cytometry was used to check cell cycle and apoptosis results. We measured the cell cycle distribution of U2OS (8.5 × 10^5^ cells/dish) and 143B (1.1 × 10^6^ cells/dish) cells treated with GO‐Y030 (0 μM, 0.5 μM, 1 μM, 2 μM, and 4 μM) for 24 h, assessing both cellular DNA content and cell counts, as previously described [17, 31]. The cells were gently fixed with 70% ethanol in ice overnight, followed by resuspension in PBS containing 50 mg/mL PI and 0.05 mg/mL RNase (Sigma, St. Louis, MO, USA). After 15 min at 37°C, cell cycle analysis was performed using a flow cytometer (Becton‐Dickinson, San Jose, CA, USA) equipped with a 488 nm argon‐ion laser.

### 2.4 Annexin V‐FITC Apoptosis Staining Assay

Annexin V, a fluorescent protein binding phosphatidylserine, detects early apoptosis by translocating membrane phospholipids to the cell surface before DNA breakdowns, unlike PI staining. U2OS (8.5 × 10^5^ cells/dish) and 143B (1.1 × 10^6^ cells/dish) cells were treated with GO‐Y030 (0 μM, 0.5 μM, 1 μM, 2 μM, and 4 μM) for 24 h in one 6‐cm dish, as previously described [18, 19]. After trypsinisation of cells and non‐viable floating cells, Annexin V‐FITC Apoptosis Detection Kit I (BD Biosciences, San Jose, CA, USA) was used according to the manufacturer's instructions. Afterward, flow cytometry with PI staining was used to analyse the cell cycle and differentiate between apoptosis and necrosis.

### 2.5 Human Apoptosis Array

Using a Human Apoptosis Array Kit (R&D Systems, Minneapolis, MN, USA), we analysed protein lysates from 2.0 × 10^6^ U2OS cells/dish and 2.5 × 10^6^ 143B cells/dish treated with vehicle or 4 μM GO‐Y030 for 24 h to investigate induced apoptosis mechanisms [32]. The kit simultaneously detected 35 apoptosis‐related proteins. Using biotinylated detection antibodies and chemiluminescent reagents, captured proteins on nitrocellulose membranes were visualised, following established methods [18, 19].

### 2.6 Protein Extraction and Western Blot Analysis

To investigate the molecular mechanisms further, U2OS (8.5 × 10^5^ cells/dish) and 143B (1.1 × 10^6^ cells/dish) were cultured in 6 cm plates for 16 h and treated with GO‐Y030 (0 μM, 0.5 μM, 1 μM, 2 μM, and 4 μM) for 24 h. Total cell lysates were prepared and subjected to Western blot analysis using specific primary antibodies against caspases 3, 8, and 9, cIAP‐1, XIAP, cleaved caspases 3, 8, and 9, as well as antibodies for phosphorylated and unphosphorylated forms of ERK1/2, JNK1/2, and p38 (Cell Signalling Technology, Danvers, MA, USA). Blots were incubated with horseradish peroxidase‐conjugated secondary antibodies, and band intensities were quantified by densitometry, as previously described [33, 34].

### 2.7 Statistical Analysis

Using one‐way analysis of variance (ANOVA), statistical analyses were conducted, followed by Tukey's post hoc test for equal sample sizes among more than two groups. Each experiment was performed in triplicate and repeated independently three or more times. Significance was set at *p* < 0.05.

## 3 Results

### 3.1 GO‐Y030 Causes Cytotoxicity in Osteosarcoma U2OS and 143B Cells

**FIGURE 1:**
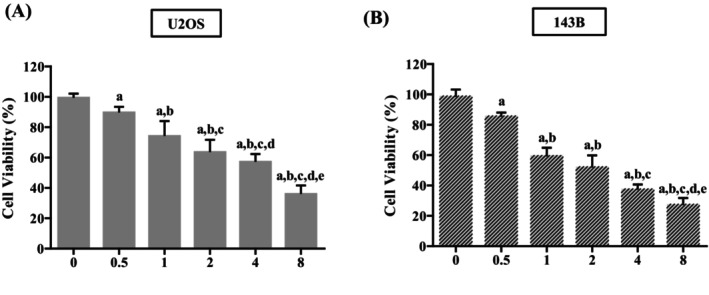
Effects of GO‐Y030 on the cell viability of U2OS and 143B cells. Using the MTT assay, the viabilities of human osteosarcoma (A) U2OS and (B) 143B cells after treatment with GO‐Y030 (0 μM, 0.5 μM, 1 μM, 2 μM, 4 μM, and 8 μM) for 24 h were detected and their effects are illustrated after quantitative analysis. Results are shown as mean ± SD ANOVA analysis with Tukey's posteriori comparison was used. U2OS: *N* = 7, *F* = 98.963, *p* < 0.001. 143B: *N* = 8, *F* = 96.067, *p* < 0.001. ^a^Significantly different, *p* < 0.05, when compared to control. ^b^Significantly different, *p* < 0.05, when compared to 0.5 μM. ^c^Significantly different, *p* < 0.05, when compared to 1 μM. ^d^Significantly different, *p* < 0.05, when compared to 2 μM. ^e^Significantly different, *p* < 0.05, when compared to 4 μM.

To verify the potency of GO‐Y030 against human osteosarcoma, we analysed the cytotoxicity of GO‐Y030 on human osteosarcoma U2OS and 143B cell lines using MTT assay. In both cell lines, viability significantly differed from controls (0 μM) with GO‐Y030 concentrations (0.5 μM, 1 μM, 2 μM, 4 μM, and 8 μM) after 24 h (*p* < 0.001 for both). The relationship showed a clear dose‐dependent trend (Figure 1B,C). Specifically, treatment with 2 μM GO‐Y030 reduced U2OS cell viability by approximately 40%, while 8 μM resulted in a 60% reduction. Similarly, 143B cells exhibited decreases of about 50% with 2 μM and 70% with 8 μM GO‐Y030 (Figure 1A,B).

### 3.2 GO‐Y030 Induces Sub‐G1 Arrest and Apoptosis in U2OS and 143 B Cells

**FIGURE 2:**
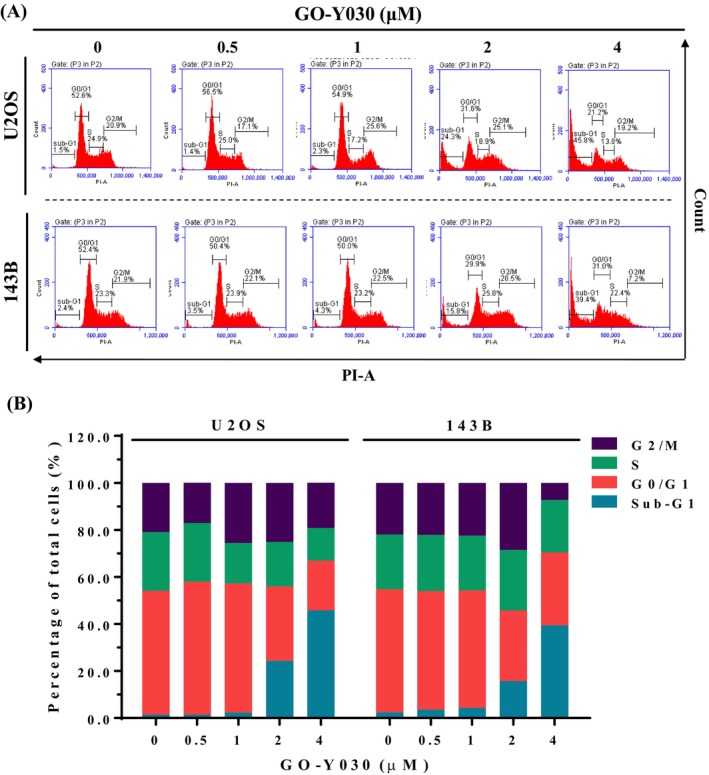
Effects of GO‐Y030 on the cell cycle of U2OS and 143B cells. (A) Using flow cytometry, U2OS and 143B were treated with GO‐Y030 (0 μM, 0.5 μM, 1 μM, 2 μM, and 4 μM) for 24 h and then subjected to analyse DNA contents after PI staining. (B) Afterward, their cell cycle profile was subsequently quantified.

Flow cytometry was used to assess cell cycle phases (G0/G1, S, G2/M) to elucidate the cytotoxic mechanism of GO‐Y030 in U2OS and 143B cells. Following 24‐h treatment with GO‐Y030 (0.5 μM, 1 μM, 2 μM, and 4 μM), a significant increase in the sub‐G1 fraction was observed: from 1.5% to 45.8% in U2OS and from 2.4% to 39.4% in 143B cells (Figure 2A,B). This indicates that cell cycle arrest in the sub‐G1 phase contributes to GO‐Y030's cytotoxic effects.

**FIGURE 3:**
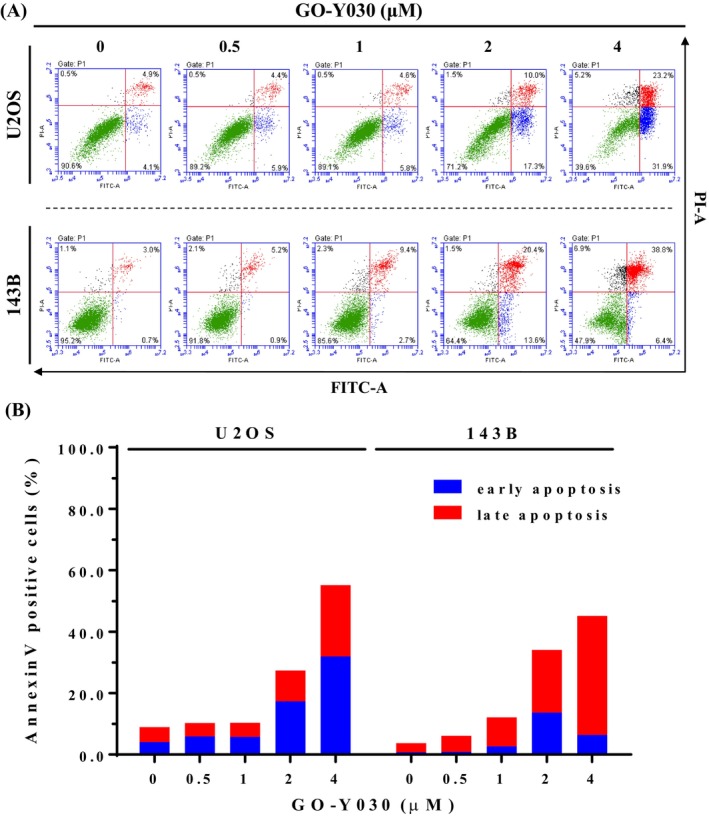
Effects of GO‐Y030 on apoptosis of U2OS and 143B cells. (A) Using flow cytometry, U2OS and 143B were treated with GO‐Y030 (0 μM, 0.5 μM, 1 μM, 2 μM, and 4 μM) for 24 h after Annexin V‐FITC/PI staining. Cells, both FITC Annexin V and PI negative, were considered viable. FITC Annexin V positive and PI negative cells were in early apoptosis. Both FITC Annexin V and PI positive cells were in late apoptosis or already dead. (B) Accordingly, quantitative analysis of early and late apoptosis was combined to distinguish apoptosis from necrosis.

To confirm whether GO‐Y030 suppresses cell growth through apoptosis rather than necrosis, we conducted an annexin V‐FITC/PI apoptosis assay on U2OS and 143B cells. After 24‐h treatment with up to 4 μM GO‐Y030 in U2OS and 143B cells, flow cytometry showed significant increases in early apoptotic cells (annexin V‐FITC positive, PI negative) and late apoptotic cells (annexin V‐FITC positive, PI positive) (Figure 3A,B). These findings indicate a marked rise in both early and late apoptotic cells (positive for annexin V‐FITC) induced by GO‐Y030, correlating with the accumulation in the sub‐G1 fraction observed in both cell lines. Consistent with the previous study [19], the curcumin analog GO‐Y030 can also potentially decrease cell viability and significantly induce apoptosis in human osteosarcoma U2OS and 143B cells.

### 3.3 GO‐Y030 Increases Cleaved Caspase 3 but Decreases cIAP‐1 and XIAP in U2OS and 143B Cells

**FIGURE 4:**
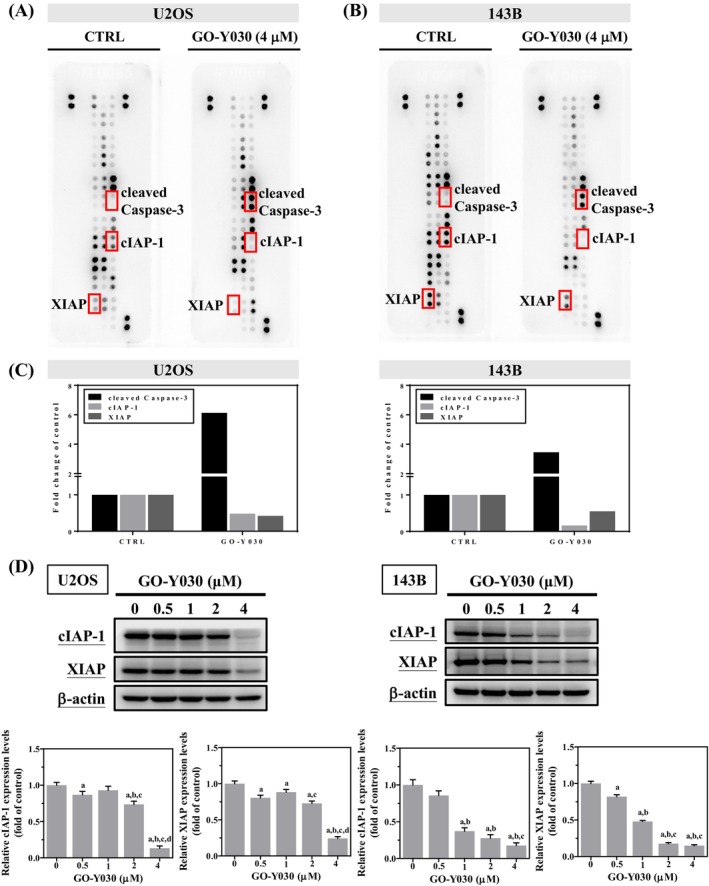
Effects of GO‐Y030 on the human apoptosis array in U2OS and 143B cells. Using the human apoptosis array as described in the Materials and Methods section, 35 apoptosis‐related proteins in (A) U2OS and (B) 143B cells were detected after treatment with 4 μM GO‐Y030 for 24 h in U2OS cells. (C) One increased and two decreased proteins were subjected to quantitative analysis. (D) After treating with GO‐Y030 (0 μM, 0.5 μM, 1 μM, 2 μM, and 4 μM) for 24 h, cIAP‐1 and XIAP expressions in U2OS and 143B cells were measured by Western blotting as described in the Materials and Methods section. Results are shown as mean ± SD *n* = 3. ANOVA analysis with Tukey's posteriori comparison was used. cIAP‐1: *F* = 191.508, *p* < 0.001. XIAP: *F* = 225.213, *p* < 0.001. ^a^Significantly different, *p* < 0.05, when compared to control. ^b^Significantly different, *p* < 0.05, when compared to 0.5 μM. ^c^Significantly different, *p* < 0.05, when compared to 1 μM. ^d^Significantly different, *p* < 0.05, when compared to 2 μM.

We used a human apoptosis array to analyse apoptosis‐related proteins and investigate the mechanisms of GO‐Y030‐induced apoptosis in U2OS and 143B cells. Treatment with 4 μM GO‐Y030 for 24 h significantly increased cleaved caspase 3 but decreased cIAP‐1 and XIAP proteins, suggesting their role as executioners in these cells (Figure 4A–C). These findings were validated by Western blotting, confirming dose‐dependent decreases in cIAP‐1 and XIAP expressions in both U2OS (*p* < 0.001 for both) and 143B cells (*p* < 0.001 for both) (Figure 4D).

### 3.4 GO‐Y030 Activates Both Extrinsic and Intrinsic Apoptotic Processes in U2OS and 143B Cells

**FIGURE 5:**
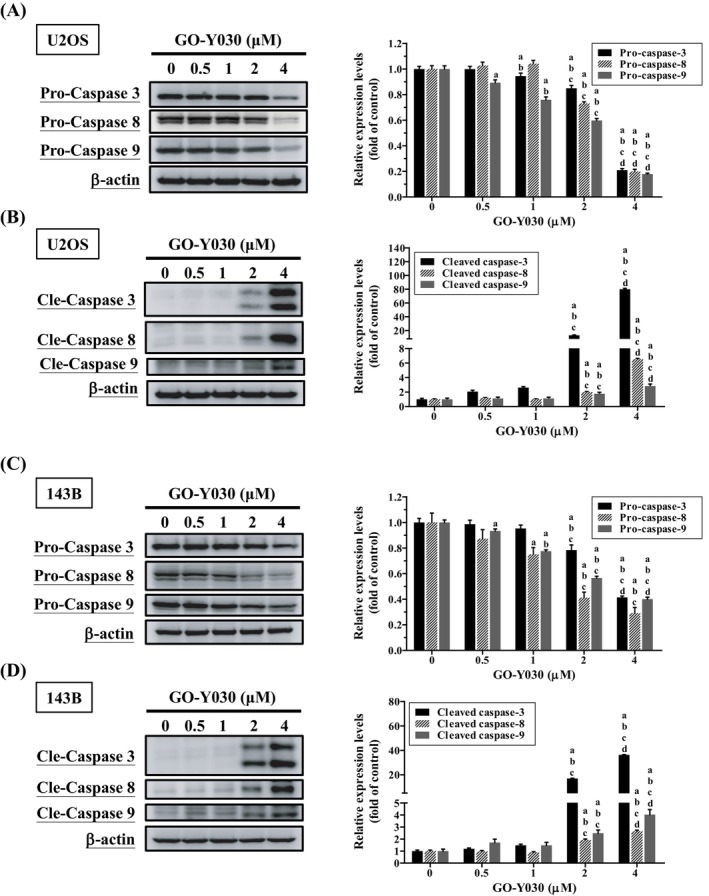
Effects of GO‐Y030 on activation of caspases 3, 8, and 9 in U2OS and 143B cells. Using Western blotting, caspases 3, 8, and 9 and their cleaved forms after treating with GO‐Y030 (0 μM, 0.5 μM, 1 μM, 2 μM, and 4 μM) for 24 h in (A, B) U2OS and (C, D) 143B cells were measured. Next, they were subjected to quantitative analysis. Results are shown as mean ± SD *n* = 3. ANOVA analysis with Tukey's posteriori comparison was used. U2OS: Pro‐caspase 3: *F* = 827.870, *p* < 0.001; pro‐caspase 8: *F* = 759.441, *p* < 0.001; pro‐caspase 9: *F* = 807.166, *p* < 0.001; cleaved caspase 3: *F* = 8174.097, *p* < 0.001; cleaved caspase 8: *F* = 2546.905, *p* < 0.001; cleaved caspase 9: *F* = 41.858, *p* < 0.001. 143B: Pro‐caspase 3: *F* = 206.487, *p* < 0.001; pro‐caspase 8: *F* = 79.429, *p* < 0.001; pro‐caspase 9: *F* = 824.104, *p* < 0.001; cleaved caspase 3: *F* = 2192.447, *p* < 0.001; cleaved caspase 8: *F* = 259.272, *p* < 0.001; cleaved caspase 9: *F* = 52.198, *p* < 0.001. ^a^Significantly different, *p* < 0.05, when compared to control. ^b^Significantly different, *p* < 0.05, when compared to 0.5 μM. ^c^Significantly different, *p* < 0.05, when compared to 1 μM. ^d^Significantly different, *p* < 0.05, when compared to 2 μM.

Using Western blotting, the effect of GO‐Y030 on the caspase cascade in U2OS and 143B cells was investigated. Treatment with increasing concentrations of GO‐Y030 for 24 h led to dose‐dependent increases in cleaved caspases 3, 8, and 9 (U2OS: *p* < 0.001 for all; 143B: *p* < 0.001 for all) (Figure 5A–D). Meanwhile, caspases 3, 8, and 9 decreased dose‐dependently. GO‐Y030 induced apoptosis in both cell lines by activating the extrinsic caspase 8 and intrinsic caspase 9 pathways, leading to downstream activation of the effector caspase 3.

### 3.5 GO‐Y030 Activates Extrinsic and Intrinsic Apoptotic Cascades via JNK and p38 Signalling in U2OS and 143B Cells

**FIGURE 6:**
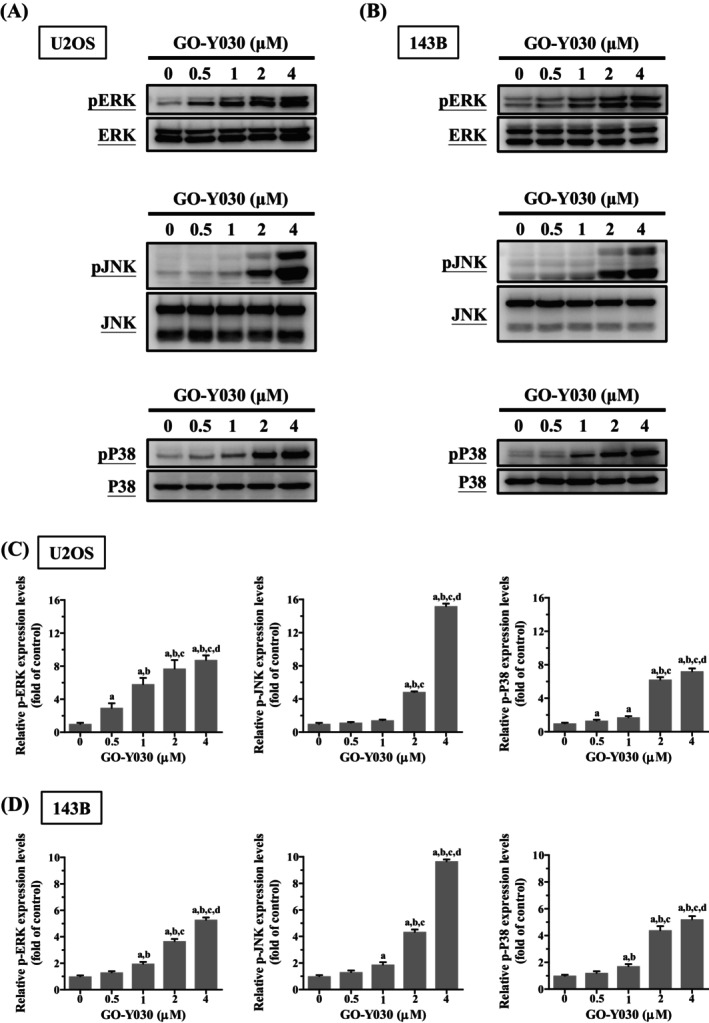
Effects of GO‐Y030 on the phosphorylation of ERK, JNK, and p38 in U2OS and 143B cells. (A, B) Using Western blotting, relative expression of ERK1/2, JNK 1/2, and p38, as well as their phosphorylation after treating with GO‐Y030 (0 μM, 0.5 μM, 1 μM, 2 μM, and 4 μM) for 24 h in U2OS and 143B cells were measured. (C, D) Subsequently, they were subjected to quantitative analysis. Results are shown as mean ± SD *n* = 3. ANOVA analysis with Tukey's posteriori comparison was used. U2OS: P‐ERK/ERK: *F* = 5155.234, *p* < 0.001; p‐JNK/JNK: *F* = 5476.686, *p* < 0.001; p‐p38/p38: *F* = 1476.949, *p* < 0.001. 143B: P‐ERK/ERK: *F* = 350.586, *p* < 0.001; p‐JNK/JNK: *F* = 748.227, *p* < 0.001; p‐p38/p38: *F* = 229.002, *p* < 0.001. ^a^Significantly different, *p* < 0.05, when compared to control. ^b^Significantly different, *p* < 0.05, when compared to 0.5 μM. ^c^Significantly different, *p* < 0.05, when compared to 1 μM. ^d^Significantly different, *p* < 0.05, when compared to 2 μM.

**FIGURE 7:**
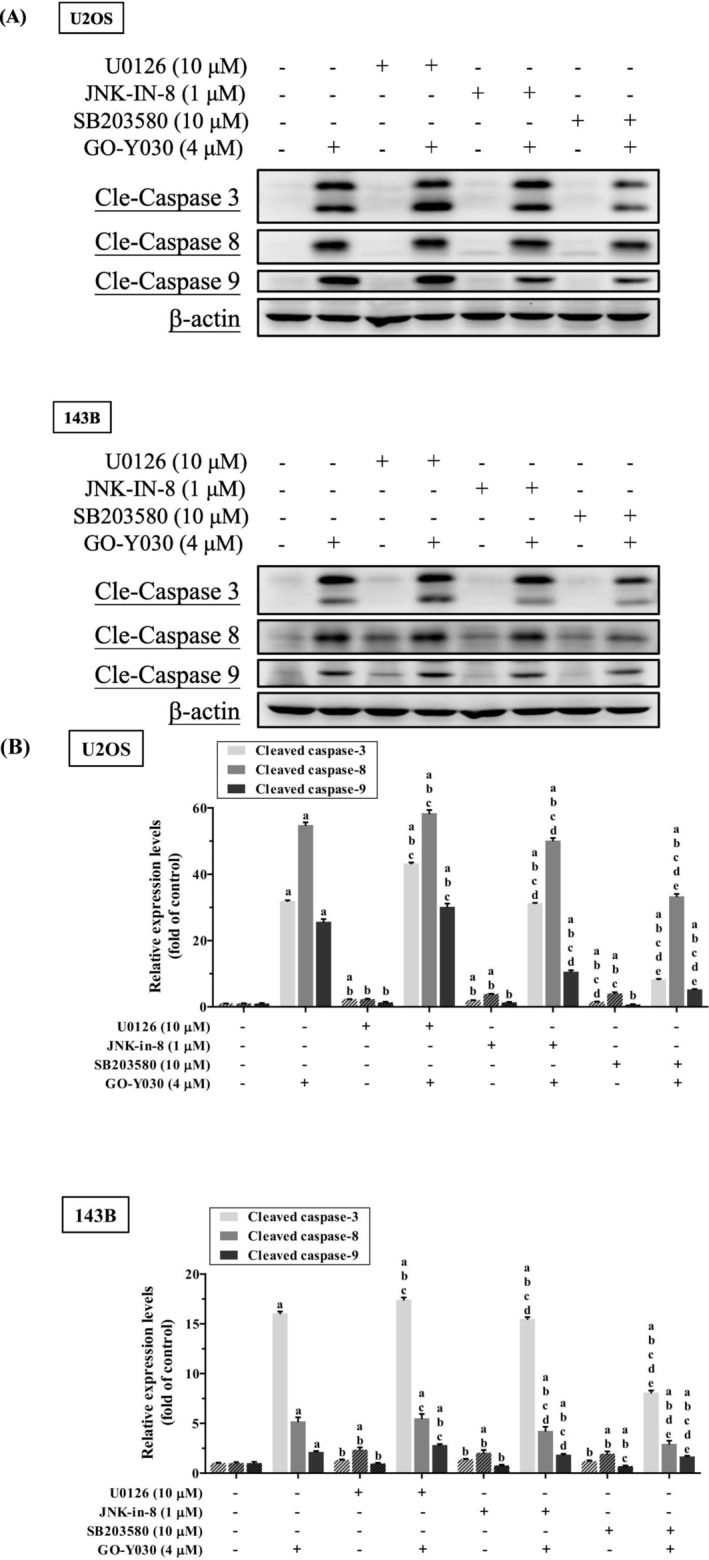
Effects of GO‐Y030 and inhibitors of ERK1/2 (U0126), JNK1/2 (JNK‐IN‐8), and p38 (SB203580) on cleaved caspases 3, 8, and 9 expressions of U2OS and 143B cells. (A) Using Western blotting, cleaved caspases 3, 8, and 9 expressions after pretreating with or without 10 μM of U0126, 1 μM of JNK‐IN‐8, and 10 μM SB203580 for 1 h, followed by 0 or 4 μM GO‐Y030 treatment for an additional 24 h in U2OS and 143B cells were measured. (B) Then, they were subjected to quantitative analysis. Results are shown as mean ± SD *n* = 3. ANOVA analysis with Tukey's posteriori comparison was used. U2OS: Cleaved caspase 3: *F* = 53440.212, *p* < 0.001; cleaved caspase 8: *F* = 5646.787, *p* < 0.001; cleaved caspase 9: *F* = 1789.565, *p* < 0.001. 143B: Cleaved caspase 3: *F* = 7323.992, *p* < 0.001; cleaved caspase 8: *F* = 79.179, *p* < 0.001; cleaved caspase 9: *F* = 205.848, *p* < 0.001. ^a^Significantly different, *p* < 0.05, when compared to control. ^b^Significantly different, *p* < 0.05, when compared to 4 μM GO‐Y030. ^c^Significantly different, *p* < 0.05, when compared to U0126. ^d^Significantly different, *p* < 0.05, when compared to JNK‐IN‐8. ^e^Significantly different, *p* < 0.05, when compared to SB203580.

Figure 6 demonstrates that GO‐Y030 dose‐dependently increased the phosphorylation of ERK1/2, JNK1/2, and p38 in both U2OS and 143B cells (U2OS: *p* < 0.001 for all; 143B: *p* < 0.001 for all), suggesting activation of these pathways by GO‐Y030. We examined the impact of ERK1/2 (U0126), JNK1/2 (JNK‐IN‐8), and p38 (SB203580) inhibitors on GO‐Y030‐induced activation of cleaved caspases 8, 9, and 3 in U2OS and 143B cells. As expected, treatment with 4 μM GO‐Y030 activated cleaved caspases 8, 9, and 3 (U2OS: *p* < 0.05 for all; 143B: *p* < 0.05 for all) (Figure 7A–D). Notably, JNK‐IN‐8 and SB203580 significantly attenuated GO‐Y030's induction of cleaved caspases 8, 9, and 3 (U2OS: JNK‐IN‐8: *p* < 0.05 for all; 143B: JNK‐IN‐8: *p* < 0.05 for all; U2OS: SB203580: *p* < 0.05 for all; 143B: SB203580: *p* < 0.05 for all), while U0126 did not ameliorate these effects; instead, it slightly enhanced them (U2OS: *p* < 0.05 for all; 143B: *p* < 0.05 for all). These results underscore the critical role of JNK and p38 signalling in GO‐Y030‐induced apoptosis via extrinsic (caspase 8) and intrinsic (caspase 9) pathways, along with their effector caspase 3, in U2OS and 143B cells.

## 4 Discussion

Curcumin and its synthetic analogs are attracting interest due to their bioactive properties, especially their anticancer effects in different cancer cell line models [18, 19, 30]. The synthesised curcumin analog GO‐Y030 was developed to enhance curcumin's bioavailability and potency, targeting cancer cell cytotoxicity while sparing normal cells. GO‐Y030 showed significantly higher growth inhibition than curcumin, with 50% growth inhibition concentrations (IC50) ranging from one‐11th to one‐14th that of curcumin [25]. It induced cell death at a 10‐fold lower concentration than curcumin and exhibited 4–15 times more growth inhibition in pancreatic and thyroid cancer cells. In both in vitro and in vivo studies, GO‐Y030 administration effectively suppressed cancer cell growth [22, 25], with its IC50 for human gastric tumour cells being 20‐fold lower than that of underivatised curcumin [20]. Additionally, GO‐Y030 exhibited 3.0 to 13.5 times larger growth‐suppressive activity than GO‐035 [20], and also demonstrated at least 10 times stronger apoptosis induction than curcumin [35], suggesting that it may be more effective for cancer therapy.

GO‐Y030 exhibits notable antitumour properties through multiple mechanisms such as apoptosis induction, regulatory T cell modulation [36], p300‐HAT [37] and mTOR‐S6 axis inhibition [36], and inhibitor of NF‐ B kinase subunit β (IKKβ, IκB kinase β) [25] and signal transducer and activator of transcription 3 (STAT3) activation blockade [24, 35, 38, 39]. Over the past decade, various developmental pathways activated by GO‐Y030 to inhibit cell growth and induce apoptosis in human cancers have been identified. These pathways include NF‐κB [25, 35], phosphoinositide 3‐kinase (PI3K)/AKT, Janus kinase (JAK)/STAT3 [24, 35, 39], and interferon regulatory factor 4 (IRF4) [35]. However, these diverse mechanisms depend on different cancer types and cell lines [1, 2, 40].

GO‐Y030 inhibits signalling pathways such as IKKβ, STAT3, and AKT, which are crucial for the proliferation and survival of multiple myeloma RPMI8226, KMS12‐BM, and OPM2 cell lines, breast cancer MDA‐MB‐231 cell line, and pancreatic carcinoma PANC‐1, HPAC, and BXPC‐3 cell lines [24, 35]. It is 7–12 times more effective at suppressing multiple myeloma cell growth and 6–15 times more potent at inhibiting the NF‐κB, PI3K/AKT, JAK/STAT3, and IRF4 pathways than curcumin [35]. Specifically, GO‐Y030 suppresses pAKTs at least 10 times stronger than curcumin. Regarding cell cycle arrest, inhibition of NF‐κB and Wnt signal transactivation, GO‐Y030 is more effective than curcumin [22]. GO‐Y030 is a strong inhibitor of NF‐κB in plasmacytoma RPMI8226 cells at least 6.4 times more than curcumin and exhibits a strong affinity of NF‐κB releasing from IκB anchoring [35]. By blocking the interaction between the HSP70/HSP40 complex and its substrates in various cancer cell lines, GO‐Y030 suppresses cancer stem cells' ability to form spheres [41]. Additionally, GO‐Y030 inhibits glycolysis, preventing lung metastasis in B16F10 melanoma cells [21].

Through various experimental practices, we found that GO‐Y030 triggers apoptotic processes via extrinsic and intrinsic pathways by activating the caspase cascade, IAPs, and phosphorylation of MAPK pathways. While phosphorylation of ERK 1/2, JNK 1/2, and p38 was observed, the use of their inhibitors confirmed that GO‐Y030 increased cleaved caspases 8, 9, and 3, but the JNK and p38 inhibitors significantly repressed these increases. This suggests the JNK and p38 signalling pathways play a critical role in apoptosis induction in osteosarcoma U2OS and 143B cell lines after GO‐Y030 treatment, unlike ERK signalling. Further studies are needed to confirm these in vivo results and the efficacy of GO‐Y030 in clinical trials for human osteosarcoma.

In conclusion, our research investigated the anticancer efficacy of GO‐Y030 and its apoptotic mechanisms in human osteosarcoma cells, suggesting its potential usefulness in adjuvant chemotherapy. In the future, it is necessary to explore novel aspects of GO‐Y030 as an adjuvant candidate, such as identifying synergistic effects in combination with other chemotherapeutic agents, to provide various treatment options for human osteosarcoma.

## Author Contributions

**Yu‐Hsien Lin:** conceptualization (equal), data curation (equal), writing – original draft (equal), writing – review and editing (equal). **Jia‐Sin Yang:** conceptualization (equal), data curation (equal), methodology (equal). **Chia‐Hsuan Chou:** methodology (equal). **Tzu‐Yu Huang:** methodology (equal). **Shun‐Fa Yang:** conceptualization (equal), data curation (equal), writing – original draft (equal), writing – review and editing (equal). **Ko‐Hsiu Lu:** conceptualization (equal), data curation (equal), writing – original draft (equal), writing – review and editing (equal).

## Conflicts of Interest

The authors declare no conflicts of interest.

## Data Availability

The data used to support the findings of this study are available from the corresponding author upon reasonable request.
